# Long Noncoding RNA Metastasis-Associated Lung Adenocarcinoma Transcript 1 in Extracellular Vesicles Promotes Hepatic Stellate Cell Activation, Liver Fibrosis and β-Catenin Signaling Pathway

**DOI:** 10.3389/fphys.2022.792182

**Published:** 2022-02-14

**Authors:** Tianqi Wang, Chong Zhang, Xiaoming Meng, Benshuai Zhu, Siyu Wang, Wenkang Yuan, Sumei Zhang, Jiegou Xu, Chao Zhang

**Affiliations:** ^1^Department of General Surgery, The First Affiliated Hospital of Anhui Medical University, Hefei, China; ^2^Inflammation and Immune Mediated Diseases Laboratory of Anhui Province, Anhui Institute of Innovative Drugs, School of Pharmacy, Anhui Medical University, Hefei, China; ^3^Department of General Surgery, Huashan Hospital, Fudan University, Shanghai, China; ^4^Department of Biochemistry and Molecular Biology, Anhui Medical University, Hefei, China; ^5^Department of Immunology, School of Basic Medical Sciences, Anhui Medical University, Hefei, China

**Keywords:** Lnc-MALAT1, hepatic stellate cells, liver fibrosis, β-catenin signaling pathway, EVS

## Abstract

Evidence shows that the long noncoding RNA metastasis-associated lung adenocarcinoma transcript 1 (Lnc-MALAT1) is associated with activation of hepatic stellate cells (HSCs) and liver fibrosis in animal and *in vitro* studies. However, its roles in human liver fibrosis and the underlying mechanism in HSC activation are not yet defined. In our current study, the expression of Lnc-MALAT1 in the fibrotic liver tissues and in the plasma extracelllar vesicles (EVs) of liver fibrosis patients was detected by FISH and qRT-PCR. The results revealed that enhanced expression of Lnc-MALAT1 was co-localized with increased expression of the fibrotic markers (collagen I and α-SMA) and the Wnt/β-catenin signaling proteins (β-catenin, cyclinD1 and c-myc) in the fibrotic liver tissues. The level of Lnc-MALAT1 in the plasma EVs isolated from 60 liver fibrosis patients was significantly increased compared with that of the 46 control patients, and area under receiver operating curve (AUROC) analysis showed that plasma EVs-Lnc-MALAT1 was a potential diagnostic marker for liver fibrosis, especially for high liver fibrosis. Plasma EVs with highly expressed Lnc-MALAT1 derived from high liver fibrosis patients up-regulated the expression of the fibrotic markers and enhanced the Wnt/β-catenin signaling in human hepatic stellate cells LX-2, and the fibrogenic effects in LX-2 were inhibited by Lnc-MALAT1 knock-down. Interestingly, TGF-β1, a potent pro-fibrotic cytokine, promoted the expression of Lnc-MALAT1 in LX-2 and its pro-fibrotic effects were also abolished by siRNA for Lnc-MALAT1, suggesting that Lnc-MALAT1 probably functions as a common mediator in the activation and fibrogenesis of HSCs. Our results indicate that enhanced expression of Lnc-MALAT1 in the fibrotic liver stimulate the activation of HSCs and thus promote their fibrogenic activity. These results also provide evidences that Lnc-MALAT1 is a potential therapeutic target and plasma EVs-Lnc-MALAT1 is a potential diagnostic biomarker for liver fibrosis.

## Introduction

Liver fibrosis is a long-term pathological process characterized by increased extracellular matrix (ECM) around the liver parenchyma (Ellis and Mann, 2012). It often occurs in response to a variety of hepatic insults such as metabolic abnormalities, hepatitis B/C virus infections, toxins, drugs, and etc., (Pu et al., 2021). When an insult induces a liver injury, the parenchymal cells regenerate to replace the injured hepatic cells. Persistent insults will result in regeneration failure of the parenchymal cells, eventually leading to increased hepatocyte apoptosis, hepatic stellate cell (HSC) activation and fibrogenesis (Zhang et al., 2017).

The mechanism of hepatic fibrogenesis is complex and HSC activation is one of the most important contributors (Lee and Friedman, 2011; Gandhi, 2017; Dewidar et al., 2019). HSCs become rapidly activated by reactive oxygen species (ROS), cytokines, growth factors and microRNAs produced during liver injury (Puche et al., 2013). Once activated, HSCs produce and deposit increasing amounts of type I and III collagen, a-smooth muscle actin (a-SMA) and fibronectin in the ECM, companied by imbalanced expression of matrix metalloproteinases and tissue inhibitor of metalloproteinases (Duarte et al., 2015). TGF-β, the most potent fibrogenic cytokine produced by several cells including activated HSCs, binds to its receptors and induce the Smad signaling pathway to promote procollagen mRNA expression (Mann and Mann, 2009). Other factors such as β-catenin, INF-g and CTGF also contribute to activation and ECM synthesis of HSCs *via* different signaling pathways (Rachfal and Brigstock, 2003; Chen et al., 2005; Monga, 2015).

Recently, emerging evidence has shown that long noncoding RNAs (lncRNAs), RNA transcripts longer than 200 nucleotides with regulatory roles in many biological processes, participate in activation and fibrogenesis of HSCs. Zhang et al. (2017) identified a lncRNA, lnc-LFAR1, in CCl_4_- and bile duct ligation-induced liver fibrosis mouse models. Lnc-LFAR1 promoted HSC activation and liver fibrosis through TGF-β and Notch pathways. Other lncRNAs including nuclear paraspeckle assembly transcript-1 (NEAT-1), Hox A distal transcript (HOTTIP), metastasis-associated lung adenocarcinoma transcript 1 (Lnc-MALAT1), highly upregulated in liver cancer (HULC), small cajal body specific RNA 10 (SCARNA10), small nuclear RNA host gene 7 (SNHG7) and plasmocytoma variant translocation 1 (PVT1) have been found to be up-regulated in fibrotic liver tissues to induce HSC activation and enhance accumulation of ECM through various mechanisms (Ganguly and Chakrabarti, 2021). Among these lncRNAs, our special interest is Lnc-MALAT1. Besides its controversial roles in liver fibrogenesis, Lnc-MALAT1 is found to be packed into EVs, suggesting that it may be used as an indicator of liver fibrosis (Dai et al., 2019).

In our current study, EVs-Lnc-MALAT1 in the plasma of liver fibrosis patients was detected to evaluate whether it had diagnostic values for liver fibrosis. Simultaneously, the role of EVs-Lnc-MALAT1 in liver fibrosis and the associated fibrogenic mechanism were investigated in the human hepatic stellate cells LX-2 *in vitro* and in human fibrotic liver tissues.

## Materials and Methods

### Patients

Plasma and tissue samples from 60 informed patients with liver fibrosis and 46 informed controls were collected from the First Affiliated Hospital of Anhui Medical University (Hefei, China) between August 2019 and June 2021 and accordance with the declaration of Helsinki. Inclusion criteria: patients in normal group are hepatitis B virus infected, diagnosed to be normal by liver biopsy and do not received treatment, while patients in fibrosis group are hepatitis B virus infected, diagnosed to be liver fibrosis by liver biopsy and do not received treatment. Exclusion criteria: alcoholic liver, non-alcoholic fatty liver disease, autoimmune hepatitis, other types of hepatitis, cirrhotic decompensation, liver cancer and other systemic fibrotic diseases. Liver fibrosis tissues were obtained from patients with liver fibrosis who undertook biopsy and hepatectomy, while the control liver tissues were collected from patients with hepatic hemangiomas or hepatic cysts who received hepatectomy. Signed informed consent was obtained from all patients before the specimen collection. Characteristics of all the patients are shown in Supplementary Table 2. Ethical approval for this study was granted by the Ethics Committee of the Faculty of Science, First Affiliated Hospital of Anhui Medical University.

### Extracelllar Vesicles Extraction and Characterization

In total samples, 4 ml of plasma were centrifuged at 3000 × *g* for 30 min to remove cellular debris and then centrifuged at 4°C, 100,000 × *g* for 90 min. The EVs pellet was resuspended in 4 ml PBS and filtered with 0.22 μm filter (Millpore, Massachusetts) centrifuged at 4°C, 100,000 × *g* for 90 min. The EVs pellet was resuspended in 50 ul PBS and stored at −80°C for later use. For observation of EVs structure, the EVs solution was loaded on a carbon-coated copper grid, washed and negatively stained with uranyl acetate, and then observed under Transmission Electron Microscopy (TEM, JEM 1,400, Japan). For analysis of the components of the isolated EVs, TSG101 and CD81 were detected by western blotting. For analysis of the size distribution, the EVs solution was diluted 1:20 in PBS and analysized using particle size analysis System (Zetasizer Nano, Malvern, United Kingdom). The protein concentration of the EVs was detected using Bicinchoninic Acid Assay (BCA, Beyotime, Shanghai).

### Cell Culture and Treatment

The immortalized human hepatic stellate cell line LX-2 was provided by Dr. Scott Friedman (Mount Sinai School of Medicine, United States) and was detected to be free of mycoplasma. LX-2 cells were cultured in Dulbecco’s modified Eagle’s medium (DMEM, Gibco, New York) supplemented with 10% fetal bovine serum (FBS, Gibco, New York), 100 U/mL penicillin and 100 U/mL streptomycin, and maintained at 37°C in a humidified environment containing 5% CO2. For EVs related experiments, Exosome-Free FBS (Absin Bioscience, Shanghai) was used to exclude the EVs contamination from fetal bovine. The culture medium was changed every 1–2 days.

For assessment of the effect of EVs isolated from the plasma of the liver fibrosis patients on LX-2 fibrogenesis, 1 × 10^6^ LX-2 cells on a 6-well plate were transfected with 0.8ug/ml of Lnc-MALAT1-specific small interference RNA (siRNA-Lnc-MALAT1, GenePharma, Shanghai) or irrelevant small RNA (as a negative control, siRNA-NC, GenePharma, Shanghai) by lipo3000 (Invitrogen, CA, United States) for 4 h. After replacement of the culture medium with fresh medium, the cells were treated with 100 ug plasma EVs from liver fibrosis patients or from the control subjects and continued to culture for another 44 h. The cells were collected for quantitative real-time-PCR (qRT-PCR) and western blot analysis. Treatment with TGF-β1 (10 ng/ml in PBS) was used as a positive control and with equivalent PBS as a negative control.

For assessment of the effect of endogenous Lnc-MALAT1 on TGF-β1-induced fibrogenesis, 1 × 10^6^ LX-2 cells on a 6-well plate were transfected with 0.8ug/ml of siRNA- Lnc-MALAT1) or siRNA-NC by lipo3000 (Invitrogen, CA, United States) for 4 h. After replacement of the culture medium with fresh medium, the cells were treated with PBS or 10ng/ml TGF-β1 for another 44 h. The cells were collected for qRT-PCR and western blot analyses.

### Western Blot

Tissues, cells and EVs were lysed with RIPA buffer (Beyotime, Shanghai) and centrifuged at 4°C, 12,000 × *g* for 30 min. The supernatants were collected and the protein concentration was determined by BCA protein assay kit. Total 20 mg proteins in the samples were separated by 10% sodium dodecyl sulfate polyacrylamide gel electrophoresis and transferred to polyvinylidene difluoride membranes (Millipore Corp, Billerica, MA, United States). After blocking in 5% skim milk, the membranes were washed with Tween 20-buffered saline (TBS) and incubated overnight with the specific primary antibodies at 4°C. Rabbit polyclonal antibodies against α-SMA (1:1000, 14395-1-AP, Proteintech, Chicago), Collagen I (1:1000, ab270993, Abcam, United States), β-catenin (1:5000, 51067-2-AP, Proteintech, Chicago), β-actin (1:5000, 20536-1-AP, Proteintech, Chicago), c-myc (1:1000, sc-40, Santa Cruz Biotechnology, Dallas), Cyclin-D1 (1:5000, ab40754, Abcam, United Kingdom), TSG101 (1:5000, ab125011, Abcam, United Kingdom), CD81 (1:5000, ab109201, Abcam, United Kingdom), and Calnexin (1:1000, ab75801, Abcam, United Kingdom) were used, respectively. The membranes were incubated with the corresponding horseradish peroxidase-conjugated secondary antibodies for 1 h at room temperature. After washing with TBS, protein bands were visualized with an ECL chemiluminescence kit (ECL-plus, Thermo Fisher, MA, United States) by a chemiluminescent western blotting detection system (chemidoc, BioRad, CA, United States). The intensity of the bands was analyzed with Image J software.

### Quantitative Real-Time PCR

Total RNA was isolated in cell, tissue and EVs samples using Trizol reagent (Invitrogen, CA, United States) according to the manufacturer’s instructions. Extracted total RNA was resuspended with RNase-free water and the concentration was measured and using Nano Drop 2,000 (Thermo Fisher, MA, United States). In total RNAs, 500 ng was reverse transcribed and then quantitively analyzed using RT reagent Kit and SYBR-Green Master Mix (Takara, Japan). Relative primer sequences are listed in Supplementary Table 1. Duplicate of each sample were determined. GAPDH was used as an internal reference.

### Fluorescence *in situ* Hybridization

Fluorescence *in situ* hybridization (FISH) was performed using a probe specifically targeting Lnc-MALAT1 (GenePharma, Shanghai) according to the manufacturer’s instructions. Briefly, paraffin sections were dewaxed with xylene and proteins were removed by proteinase K. After heat denaturation, the sections were incubated overnight at 37°C with the probe (gacggttgagaagtggcaaaatatagcgtgtggaaagatttgagtgagggaggcaaaaagtagttc, 1 μmol/L) target Lnc-MALAT1, then subjected to DAPI staining for the nucleus and finally observed under a fluorescent microscope (BX60, Olympus, Japan).

### Hematoxylin-Eosin Staining, Masson Staining, and Immunohistochemistry

Liver tissues from liver fibrosis patients and the control were formalin-fixed and paraffin-embedded. Hematoxylin-eosin (HE) staining and Masson staining were performed according to a standard procedure and used to evaluate the changes in liver pathology and collagen deposition, respectively. Liver fibrosis patients were divided into high fibrosis score group (*s* = 3–4) and low fibrosis scores group (*s* = 1–2) by two pathologists independently according to the Ishak scoring (kappa = 0.8453)(Ishak et al., 1995).

For immunohistochemistry (IHC), the slides were treated with 0.3% hydrogen peroxide, blocked in 2% bovine serum albumin. Primary antibodies against α-SMA (1:100, 14395-1-AP, Proteintech, Chicago, IL, United States), Collagen I (1:250, ab270993, Abcam, United Kingdom), β-catenin (1:100, 51067-2-AP, Proteintech, Chicago, IL, United States), c-myc (1:100, sc-40, Santa Cruz Biotechnology, Dallas, TX, United States) and Cyclin-D1 (1:250, ab40754, Abcam, United Kingdom) were used for incubation overnight at 4°C. The sections were then incubated with biotinylated secondary antibody at room temperature for 1 h. All the slides were observed under a light microscope (DM6B, Leica, Germany) and assessed by using the software of Image-pro plus 6.0.

### Statistical Analysis

Area under curve (AUC) and 95% confidence interval were used to assess the diagnostic efficiency of plasma EVs-Lnc-MALAT1 for detecting liver fibrosis. The Youden indices were used to confirm the optimal cutoff values and their best sensitivity and specificity combination in the training set. The level of Lnc-MALAT1 in EVs were expressed as medians with interquartile ranges, and the remaining data are expressed as mean ± SD (standard deviation). The student’s *t*-test was used to compare differences between groups. When the data were not normally distributed, we used the Mann-Whitney *U*-test for data analysis. Associations between gender were analyzed using 2-tailed χ2 testing. *P*-values less than 0.05 were considered to be significantly different. All statistical analyses were performed with Graph pad Prism 8.0.

## Results

### Long Noncoding RNA Metastasis-Associated Lung Adenocarcinoma Transcript 1 Correlates With Liver Fibrosis

The liver tissues from 60 liver fibrosis patients, together with 46 patients with hepatic hemangiomas or hepatic cysts as the control liver tissues, were first subjected for HE staining and IHC analysis. Obvious fibrotic liver tissues were observed in the liver fibrosis patients (Supplementary Figure 1A). Also, enhanced expression of the typical markers Collagen I and a-smooth muscle actin (a-SMA) was detected by IHC (Supplementary Figure 1B), and relative integral optical density (IOD) of collagen I and a-SMA staining was significantly increased, compared with those in the control liver tissues (Supplementary Figure 1C). Similarly, western blotting and qPCR analyses indicated that the protein and mRNA expression of Collagen I and a-SMA was up-regulated in the fibrotic liver tissues (Supplementary Figures 1D–E). Other clinical characteristics of the 106 patients were summarized in the Supplementary Table 2.

**FIGURE 1:**
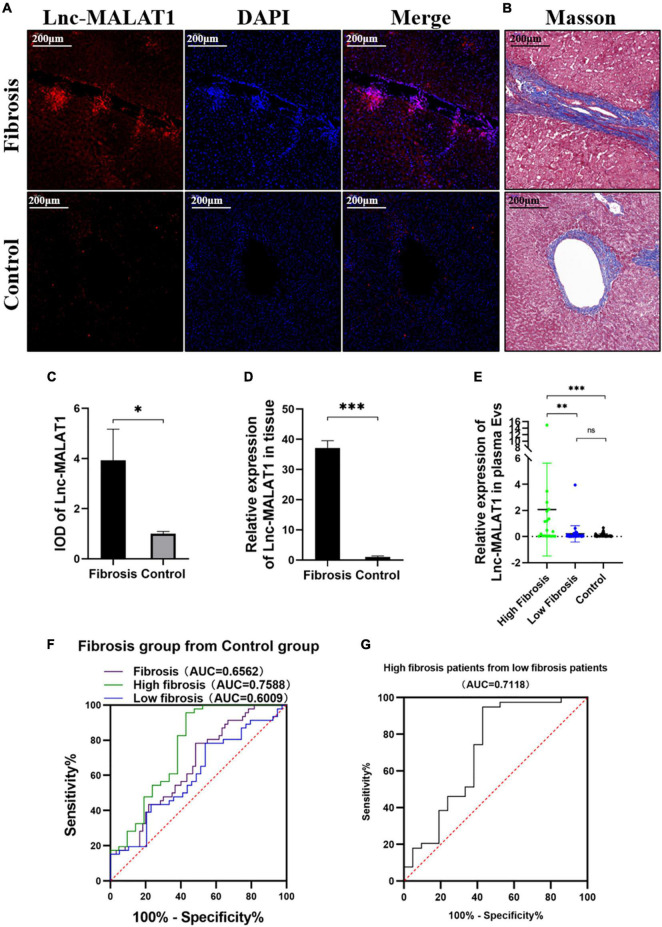
Expression of Lnc-MALAT1 in the fibrotic liver tissues and plasma EVs-Lnc-MALAT1 as a potential diagnostic marker. **(A)** Representative image of fluorescence *in situ* hybridization (FISH) targeting Lnc-MALAT1 in the liver specimens from the high fibrosis group and the control group; **(B)** Representative image of Masson staining in the consecutive tissue slides. **(C)** Relative integral optical density (IOD) value of Lnc-MALAT1 measured by Image-pro plus (*n* = 6 in each group. **(D)** Levels of Lnc-MALAT1 in the liver tissues determined by qRT-PCR (*n* = 6 in each group). **(E)** Scatter plot of the Lnc-MALAT1 expression in plasma EVs derived from the high fibrosis group (*n* = 21), low fibrosis group (*n* = 39) and control group (*n* = 46). **(F)** ROC analysis of the EVs-Lnc-MALAT1 level as a diagnostic marker for liver fibrosis. **(G)** ROC analysis of the EVs-Lnc-MALAT1 level for discrimination of high fibrosis from low fibrosis. *, **, and *** represent *p-*values less than 0.05, 0.01, and 0.001, respectively.

For observation of Lnc-MALAT1 expression in the fibrotic liver tissues, the consecutive sections of the liver tissues were used for FISH and Masson staining. Lnc-MALAT1 was found to be enriched in the liver tissues of the liver fibrosis patients compared to in the liver tissues of the control patients, and mostly located within and surrounding the fibrotic lesions (Figures 1A,B). Quantitive analysis of the FISH-stained sections revealed that IOD in the liver tissues of liver fibrosis patients was much higher than those of the controls (Figure 1C). Similar results were obtained by qPCR detection of Lnc-MALAT1 expression in the liver tissues (Figure 1D). These results suggest that Lnc-MALAT1 expression is associated with liver fibrogenesis.

### Long Noncoding RNA Metastasis-Associated Lung Adenocarcinoma Transcript 1 in Plasma Extracelllar Vesicles Was a Potential Indicator of Liver Fibrosis

To find out whether Lnc-MALAT1 exists in EVs, we first isolated the EVs from the plasma samples of the patients and then determined Lnc-MALAT1 by qRT-PCR. The level of lnc-MALAT1 is 0.8589 (± 2.3145) in fibrosis group and 0.0887 (± 0.1299). Characterization of the isolated EVs, including TEM observation of shape and structure, western blotting identification of EVs-components, and Nanoparticle tracking Analysis (NTA) size distribution, was presented in Supplementary Figure 2. TSG101 and CD81 were highly enriched in plasma EVs but were barely detectable in the whole-cell lysates (Supplementary Figure 2C).

For analysis of correlation of the plasma EVs-Lnc-MALAT1 with liver fibrogenesis, the 60 liver fibrosis patients were further divided into a high fibrosis group (21 patients, scoring = 3–4) and a low fibrosis group (39 patients, scoring = 1–2) according to the pathological Ishak scoring. Detection by qRT-PCR showed that relative expression of plasma EVs-Lnc-MALAT1 was 0.089, 0.208, and 2.068 in the control, the low fibrosis and the high fibrosis patients, respectively, showing that relative expression of plasma EVs-Lnc-MALAT1 was correlated with liver fibrogenesis (Figure 1E). Therefore, the diagnostic value of the plasma EVs-Lnc-MALAT1 in liver fibrosis was evaluated by ROC analysis. AUCs in the whole fibrosis patients, high fibrosis patients and low fibrosis group were 0.656, 0.759, and 0.601 (Figure 1F). The high fibrosis group had the highest AUC, with the sensitivity and specificity of 0.571 and 0.957, respectively. In addition, the AUC in discriminating the high fibrosis group from the low fibrosis group was 0.712 (Figure 1G). More detailed results of the ROC analysis including *p-*values and critical values were shown in Table 1. These data suggest that plasma EVs-Lnc-MALAT1 is a potential indicator of liver fibrosis, although further investigations are required.

**TABLE 1:** Diagnostic values of Lnc-MALAT1 for liver fibrosis.

	Medium	AUC	95%CI	*p*-value	Youden index	Critical value	Sensitivity	Specificity
Fibrosis vs. Control	0.050130	0.6562	0.5532–0.7591	0.0264	0.299275362	0.0749695	0.516667	0.782609
High fibrosis vs. Control	0.049003	0.7588	0.6201–0.8975	<0.001	0.527950311	0.3682010	0.571429	0.956522
Low fibrosis vs. Control	0.045663	0.6009	0.4798–0.7220	0.2122	0.244147157	0.0749695	0.461538	0.782609
High fibrosis vs. Low fibrosis	0.076662	0.7118	0.5584–0.8653	0.0023	0.520146520	0.3632260	0.571429	0.948718

### The β-Catenin Signaling Pathway Is Enhanced in the Fibrotic Liver Tissues and Activated Hepatic Stellate Cells

**FIGURE 2:**
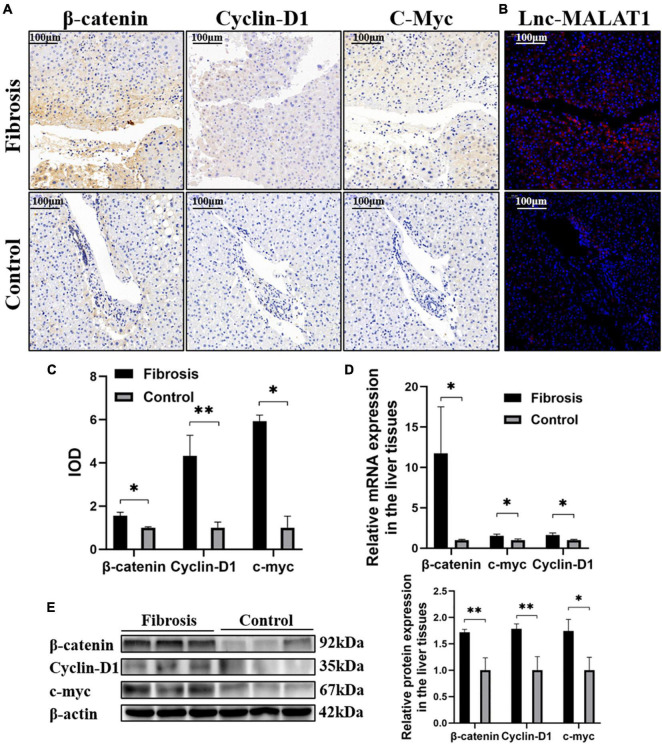
Correlation of enhanced β-catenin signaling with up-regulated Lnc-MALAT1 expression in the fibrotic liver tissues. The liver tissue specimens from the high fibrosis group and control group (*n* = 6 in each group) were used for IHC, FISH and qRT-PCR analyses. **(A)** Representative images of IHC detection of the expression of β-catenin, cyclin-D1, and c-myc. **(B)** Representative image of FISH for Lnc-MALAT1 in the consecutive tissue slides. **(C)** IOD values of the expression of β-catenin, cyclin-D1, and c-myc measured by the software of Image-pro plus 6.0. **(D)** The mRNA expression of β-catenin, Cyclin-D1 and c-myc detected by qRT-PCR. **(E)** Western blot analysis of the protein expression of β-catenin, Cyclin-D1, and c-myc (three specimens from each group were detected, and relative expression intensities were expressed on the right). *, **, and *** represent *p-*values less than 0.05, 0.01, and 0.001, respectively.

Due to its etiological diversity, liver fibrogenesis has been reported to involve several signaling pathways (Ge et al., 2014; Dropmann et al., 2016). In the current study, the β-catenin signaling pathway is studied in liver fibrosis. IHC Detection revealed that the expression of β-catenin, Cyclin-D1 and c-myc, representative molecules in the β-catenin signaling pathway, was elevated in the fibrotic liver tissues, compared with those of the control liver tissues (Figures 2A,C). Lnc-MALAT1 detected by FISH in the consecutive slides was located at the same area positive for the β-catenin signaling molecules (Figure 2B). Consistent with the IHC results, qRT-PCR and western blot analyses showed that the mRNA and protein expression of β-catenin, Cyclin-D1 and c-myc was significantly higher in the fibrotic liver tissues than those in the control liver tissues (Figures 2D,E).

Activation of HSCs is an important contributor in liver fibrosis (Lang and Brenner, 1999; Zhou et al., 2008). For understanding the β-catenin signaling in activation and fibrogenesis of HSCs, LX-2 cells, a human hepatic stellate cell line, were treated with TGF-β1 to induce activation of the cells. As expected, treatment with TGF-β1 induced the expressions of α-SMA and Collagen I at both mRNA and protein levels examined by western blotting and qRT-PCR (Supplementary Figures 3A,B). The β-catenin signaling molecules β-catenin, cyclin-D1 and c-myc were also up-regulated by TGF-β1 (Supplementary Figures 3A,B). Interestingly, enhanced expression of Lnc-MALAT1 was detected in the TGF-β1-treated LX-2 cells (Supplementary Figure 3C). These results indicate that TGF-β1 activates HSCs, enhances their fibrogenic ability and increases β-catenin signaling, and also suggest that up-regulated Lnc-MALAT1 may participate in the fibrogenesis of HSCs.

### Extracelllar Vesicles Isolated From Liver Fibrosis Patients Enhanced Fibrogenesis *in vitro* in a Long Noncoding RNA Metastasis-Associated Lung Adenocarcinoma Transcript 1-Dependent Manner

**FIGURE 3:**
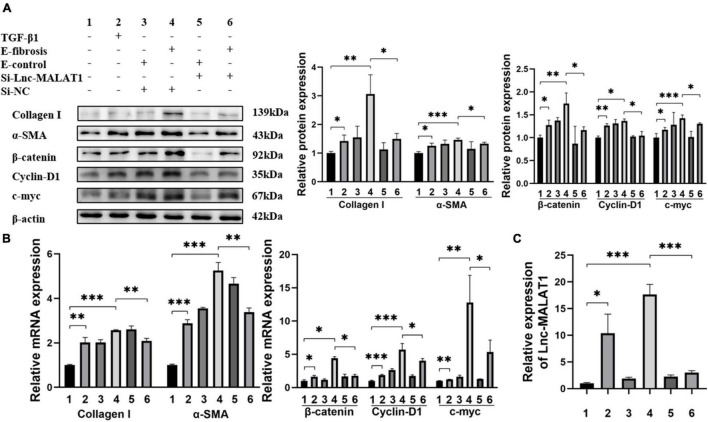
Enhanced LX-2 activation and β-catenin signaling by EVs with highly expressed Lnc-MALAT1. LX-2 cells were transfected with Lnc-MALAT1-specific siRNA or irrelevant small RNA and then treated with plasma EVs from high liver fibrosis patients or from the control subjects and continued to culture for another 44 h. The cells were collected qRT-PCR and western blot analyses. Treatment with TGF-β1 (10 ng/ml in PBS) was used as the positive control. **(A)** Western blot analysis of collagen I, α-SMA, β-Catenin, Cyclin-D1, and c-myc. The numbers indicate different treatment. Relative intensity of the bands was expressed in the middle and right panels (*n* = 6). **(B)** qRT-PCR analysis of the mRNA expression of collagen I, α-SMA, β-Catenin, Cyclin-D1 and c-myc (*n* = 6). **(C)** qRT-PCR analysis of the expression of Lnc-MALAT1 (*n* = 6). *, **, and *** represent *p-*values less than 0.05, 0.01 and 0.001, respectively.

For elucidation of the role of EVs-Lnc-MALAT1 in liver fibrosis, LX-2 cells were incubated with the EVs isolated from the plasma of patients in the high fibrosis group or from the control plasma, with TGF-β1 as a positive control. Similar to TGF-β1 (Lane 2, Figures 3A,B), the plasma EVs isolated from the high liver fibrosis patients induced increased expressions of α-SMA, Collagen I, β-catenin, cyclin-D1 and c-myc at both mRNA and protein levels in LX-2 cells (Lane 4, Figures 3A,B), as compared with those of the plasma EVs isolated from the control patients (Lane 3, Figures 3A,B). Notably, pre-transfection of siRNA specific for Lnc-MALAT1 into the cells inhibited the fibrosis plasma EVs-induced expressions of these molecules (Lane 6, Figures 3A,B), demonstrating that the enhanced effects by the plasma EVs from the liver fibrosis patients were Lnc-MALAT1-depentent. Lnc-MALAT1 expressions in the LX-2 cells with the different treatments were correlated with the enhanced effects (Figure 3C), further confirming that Lnc-MALAT1 played a pivotal role in the activation and fibrogenesis in HSCs.

### Knock-Down of Endogenous Long Noncoding RNA Metastasis-Associated Lung Adenocarcinoma Transcript 1 Inhibited TGF-β1-Induced Fibrogenesis and the β-Catenin Signaling Pathway *in vitro*

**FIGURE 4:**
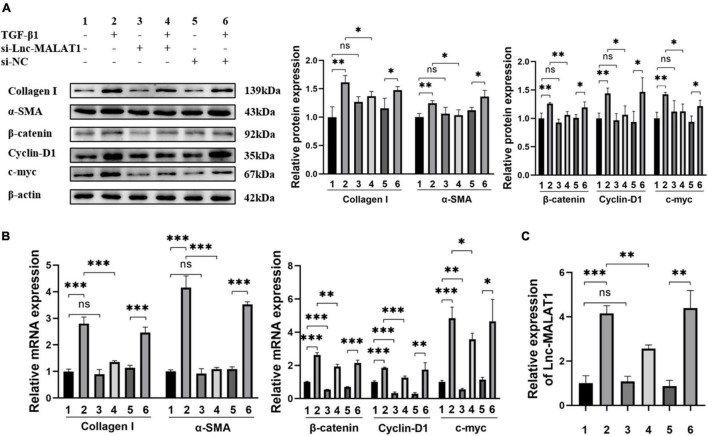
Inhibition of TGF-β1-induced fibrogenesis and β-catenin signaling in LX-2 cells by Lnc-MALAT1 knock-down. LX-2 cells were transfected with Lnc-MALAT1-specific siRNA or irrelevant small RNA, then treated with PBS or 10ng/ml TGF-β1 and continued to culture for another 44 h. **(A)** Western blot analysis of collagen I, α-SMA, β-Catenin, Cyclin-D1, and c-myc. The numbers indicate different treatment. Relative intensity of the bands was expressed in the middle and right panels (*n* = 6). **(B)** qRT-PCR analysis of the mRNA expression of collagen I, α-SMA, β-Catenin, Cyclin-D1 and c-myc (*n* = 6). **(C)** qRT-PCR analysis of the expression of Lnc-MALAT1 (*n* = 6). *, **, and *** represent *p-*values less than 0.05, 0.01 and 0.001, respectively.

The results describe above indicated that TGF-β1 induced the activation and fibrogenesis and unexpectedly, up-regulation of Lnc-MALAT1 expression in LX-2 cells. For further understanding whether the TGF-β1-induced activation and fibrogenesis depend on increased Lnc-MALAT1 expressions, we first transfected the si-RNA specific for Lnc-MALAT1 (si-Lnc-MALAT1) into LX-2 cells and then observed the fibrogenic effects of treatment with TGF-β1. Similar to the results of Supplementary Figure 3, TGF-β1 treatment enhanced the mRNA and protein expressions of α-SMA, collagen I, cyclin-D1 and c-myc into LX-2 cells (Figures 4A,B). Pre-transfection of si-Lnc-MALAT1, but not irrelevant RNA (si-NC), suppressed the TGF-β1-induced fibrogenic effects (Figures 4A,B). Consistent with these results were the expression levels of Lnc-MALAT1 as detected by qRT-PCR (Figure 4C). These observations indicate that TGF-β1-induced activation and fibrogenesis of LX-2 cells are *via* up-regulation of endogenous Lnc-MALAT1 expression.

## Discussion

Liver fibrosis is a continuous wound-healing process, triggered and sustained by pro-fibrotic cytokines such as TGF-β1 and associated with increasing in Collagen I and α-SMA expression (Zhang et al., 2017; Brandon-Warner et al., 2018). HSCs are primary targets in liver fibrosis. In response to injury and inflammation, activated HSCs secrete the extracellular matrix proteins that form fibrous scar (Popov and Schuppan, 2009; Kisseleva, 2017).

Growing number of studies have shown that Lnc-MALAT1 is associated with different kind of organ fibrosis (Leti et al., 2017; Liu et al., 2019; Huang et al., 2021). Choi et al. (2010) have reported that downregulation of Lnc-MALAT1 can decrease the level of Rac1 and thus inhibit HSC activation, while other reports indicate that Lnc-MALAT1 is an HSC activator in several animal and *in vitro* studies (Dai et al., 2019). However, the association of Lnc-MALAT1 with liver fibrosis in humans have not yet been understood. In our current research, a significantly increased expression of Lnc-MALAT1 was found in the fibrotic liver tissues and plasma EVs of liver fibrosis patients by qRT-PCR. Meanwhile, the location of Lnc-MALAT1 and Collagen I and α-SMA were detected by FISH and IHC analysis, respectively, which indicated that Lnc-MALAT1 was mainly localized in/surrounding the fibrotic tissues, suggesting a close relationship between liver fibrosis and Lnc-MALAT1. EVs isolated from the plasma of patients with high liver fibrosis promoted fibrogenic activity of the human hepatic stellate cells LX-2, as manifested by increased expression of collagen I and α-SMA. Since EVs derived from control group have stronger activated effect on the LX-2 than TGF-β1, the fibrosis process could be attributed not only to EVs-Lnc-MALAT, but also to HBV or other molecules in EVs such as mRNAs, non-coding RNAs and proteins, which need further investigation (Grant et al., 2011). The promotion effect of the isolated EVs on LX-2 was inhibited by pre-transfection of Lnc-MALAT1-specific siRNA, indicating that Lnc-MALAT1 mediated the fibrogenic activity of LX-2 cells. Our results is consistent with the finding by Dai et al. (2019) who reported that EVs with highly expressed Lnc-MALAT1 stimulated LX-2 activation. It is interesting that TGF-β1, a potent pro-fibrotic cytokine, induced Lnc-MALAT1 expression in LX-2 cells and the pro-fibrotic effect of TGF-β1 was abolished by Lnc-MALAT1-specific siRNA. It is likely that Lnc-MALAT1 is a common mediator in the fibrogenesis of hepatic stellate cells, although the underlying molecular mechanisms is still elusive.

Wnt/β-catenin signaling has been widely reported to be implicated in the pathogenesis of several liver diseases (Kikuchi and Yamamoto, 2008; Zhang et al., 2013). Our previous study demonstrated that octreotide, an analog of somatostatin, significantly inhibited CCl_4_-induced hepatic fibrosis and TGF-β1-induced activation of LX-2 cells through suppression of the Wnt/β-catenin signaling pathway (Zhang et al., 2018). In our present study, the expression of the Wnt/β-catenin signaling proteins β-catenin, cyclinD1 and c-myc, as detected by IHC staining, was up-regulated, and co-localized with Lnc-MALAT1 as detected by FISH in the fibrotic liver tissues. The expression of Wnt/β-catenin signaling molecules was enhanced in the EVs- or TGF-β1-activated LX-2 cells, which was inhibited by Lnc-MALAT1-specific siRNA. These results demonstrate that Lnc-MALAT1 has an enhancing role in the Wnt/β-catenin signaling, although the molecular mechanism needs further study.

Currently, liver biopsy and histological examination is still a main diagnostic method for liver fibrosis. Due to invasiveness of liver biopsy, non-invasive methods such as transient elastography (Lai and Afdhal, 2019) and serological determination of biomarkers including hyaluronic acid, collagen IV, procollagen type III and laminin (Liu et al., 2016) become prospective in recent years. ROC analysis of the plasma EVs-Lnc-MALAT1 levels in 60 cases of liver fibrosis patients and 46 control subjects in our current study revealed 65.6% of AUC, with 51.7% of sensitivity and 78.3% of specificity. When only patients with high fibrosis scoring were included, the diagnostic value was improved, with 75.9, 57.1, and 95.7% of the AUC, sensitivity and specificity, respectively. Interestingly, the AUC of high fibrosis group was higher than PCIII (AUC = 0.752) and the other three serological fibrosis indicators, according to the data researched by Liu et al. (Liu et al., 2016). These results indicate that plasma EVs-Lnc-MALAT1 is a potential biomarker for liver fibrosis, especially for high liver fibrosis, although the sample size was small and plasma Lnc-MALAT1 and other biomarkers were not included in the current study.

## Conclusion

Our current study revealed that enhanced expression of Lnc-MALAT1 was co-localized with increased expression of the fibrotic markers (collagen I and α-SMA) as well as the Wnt/β-catenin signaling proteins (β-catenin, cyclinD1 and c-myc) in the fibrotic liver tissues. Similar to TGF-β1, EVs with highly expressed Lnc-MALAT1 that were derived from the plasma of liver fibrosis patients up-regulated the expression of the fibrotic markers and enhanced the Wnt/β-catenin signaling in HSCs, and blocking of Lnc-MALAT1 expression by Lnc-MALAT1-specific siRNA abolished these effects in both the EVs-treated and TGF-β1-treated HSCs. As TGF-β1 stimulated the expression of Lnc-MALAT1 in HSCs, Lnc-MALAT1 probably contributes to the activation of HSCs. These results suggested that Lnc-MALAT1 is a potential therapeutic target for liver fibrosis. Another finding in our present study is that plasma EVs-Lnc-MALAT1 may be used as a diagnostic biomarker for liver fibrosis.

## Data Availability Statement

The raw data supporting the conclusions of this article will be made available by the authors, without undue reservation.

## Ethics Statement

The studies involving human participants were reviewed and approved by the Ethics Committee of the Faculty of Science, First Affiliated Hospital of Anhui Medical University. The patients/participants provided their written informed consent to participate in this study.

## Author Contributions

ChaZ and ChoZ: study design. TW: experiment conduction. BZ: collection of clinical samples and data. SW and WY: statistical analysis. TW and XM: draft. JX and SZ: manuscript revision. All authors have approved the final manuscript.

## Conflict of Interest

The authors declare that the research was conducted in the absence of any commercial or financial relationships that could be construed as a potential conflict of interest.

## Publisher’s Note

All claims expressed in this article are solely those of the authors and do not necessarily represent those of their affiliated organizations, or those of the publisher, the editors and the reviewers. Any product that may be evaluated in this article, or claim that may be made by its manufacturer, is not guaranteed or endorsed by the publisher.

## References

[B1] Brandon-Warner E. Benbow J. H. Swet J. H. Feilen N. A. Culberson C. R. McKillop I. H. (2018). Adeno-associated virus serotype 2 vector-mediated reintroduction of microrna-19b attenuates hepatic fibrosis. *Hum. Gene Therapy* 29 674–686. 10.1089/hum.2017.035 29281894 PMC6004090

[B2] Chen M. Wang G. J. Diao Y. Xu R. A. Xie H. T. Li X. Y. (2005). Adeno-associated virus mediated interferon-gamma inhibits the progression of hepatic fibrosis in vitro and in vivo. *World J. Gastroenterol.* 11 4045–4051. 10.3748/wjg.v11.i26.4045 15996030 PMC4502101

[B3] Choi S. S. Witek R. P. Yang L. Omenetti A. Syn W. K. Moylan C. A. (2010). Activation of Rac1 promotes hedgehog-mediated acquisition of the myofibroblastic phenotype in rat and human hepatic stellate cells. *Hepatology* 52 278–290. 10.1002/hep.23649 20578145 PMC2920128

[B4] Dai X. Y. Chen C. Xue J. C. Xiao T. Mostofa G. Wang D. P. (2019). Exosomal MALAT1 derived from hepatic cells is involved in the activation of hepatic stellate cells via miRNA-26b in fibrosis induced by arsenite. *Toxicol. Lett.* 316 73–84. 10.1016/j.toxlet.2019.09.008 31513886

[B5] Dewidar B. Meyer C. Dooley S. Meindl-Beinker N. (2019). TGF-beta in hepatic stellate cell activation and liver fibrogenesis-updated 2019. *Cells* 8:1419. 10.3390/cells8111419 31718044 PMC6912224

[B6] Dropmann A. Dediulia T. Breitkopf-Heinlein K. Korhonen H. Janicot M. Weber S. N. (2016). TGF-beta 1 and TGF-beta 2 abundance in liver diseases of mice and men. *Oncotarget* 7 19499–19518. 10.18632/oncotarget.6967 26799667 PMC4991397

[B7] Duarte S. Saber J. Fujii T. Coito A. J. (2015). Matrix metalloproteinases in liver injury, repair and fibrosis. *Matrix Biol.* 44-46 147–156. 10.1016/j.matbio.2015.01.004 25599939 PMC4495728

[B8] Ellis E. L. Mann D. A. (2012). Clinical evidence for the regression of liver fibrosis. *J. Hepatol.* 56 1171–1180. 10.1016/j.jhep.2011.09.024 22245903

[B9] Gandhi C. R. (2017). Hepatology snapshot: hepatic stellate cell activation and pro-fibrogenic signals. *J. Hepatol.* 67 1104–1105. 10.1016/j.jhep.2017.06.001 28939135 PMC5679016

[B10] Ganguly N. Chakrabarti S. (2021). Role of long non-coding RNAs and related epigenetic mechanisms in liver fibrosis (Review). *Int. J. Mol. Med.* 47:04856. 10.3892/ijmm.2021.4856 33495817 PMC7846421

[B11] Ge W. S. Wang Y. J. Wu J. X. Fan J. G. Chen Y. W. Zhu L. (2014). beta-catenin is overexpressed in hepatic fibrosis and blockage of Wnt/beta-catenin signaling inhibits hepatic stellate cell activation. *Mol. Med. Rep.* 9 2145–2151. 10.3892/mmr.2014.2099 24691643 PMC4055486

[B12] Grant R. Ansa-Addo E. Stratton D. Antwi-Baffour S. Jorfi S. Kholia S. (2011). A filtration-based protocol to isolate human plasma membrane-derived vesicles and exosomes from blood plasma. *J. Immunol. Methods* 371 143–151. 10.1016/j.jim.2011.06.024 21741384

[B13] Huang H. Z. Zhang G. W. Ge Z. Y. (2021). lncRNA MALAT1 promotes renal fibrosis in diabetic nephropathy by targeting the miR-2355-3p/IL6ST Axis. *Front. Pharmacol.* 12:647650. 10.3389/fphar.2021.647650 33995063 PMC8117091

[B14] Ishak K. Baptista A. Bianchi L. Callea F. De Groote J. Gudat F. (1995). Histological grading and staging of chronic hepatitis. *J. Hepatol.* 22 696–699. 10.1016/0168-8278(95)80226-6 7560864

[B15] Kikuchi A. Yamamoto H. (2008). Tumor formation due to abnormalities in the beta-catenin-independent pathway of Wnt signaling. *Cancer Sci.* 99 202–208. 10.1111/j.1349-7006.2007.00675.x 18271916 PMC11159738

[B16] Kisseleva T. (2017). The origin of fibrogenic myofibroblasts in fibrotic liver. *Hepatology* 65 1039–1043. 10.1002/hep.28948 27859502 PMC5476301

[B17] Lai M. Afdhal N. H. (2019). Liver fibrosis determination. *Gastroenterol. Clin. North Am.* 48 281–289. 10.1016/j.gtc.2019.02.002 31046975

[B18] Lang A. Brenner D. A. (1999). Gene regulation in hepatic stellate cell. *Italian J. Gastroenterol. Hepatol.* 31 173–179. 10363203

[B19] Lee U. E. Friedman S. L. (2011). Mechanisms of hepatic fibrogenesis. *Best Pract. Res. Clin. Gastroenterol.* 25 195–206. 10.1016/j.bpg.2011.02.005 21497738 PMC3079877

[B20] Leti F. Legendre C. Still C. D. Chu X. Petrick A. Gerhard G. S. (2017). Altered expression of MALAT1 lncRNA in nonalcoholic steatohepatitis fibrosis regulates CXCL5 in hepatic stellate cells. *Transl. Res.* 190 25–39. 10.1016/j.trsl.2017.09.001 28993096 PMC5705449

[B21] Liu B. Qiang L. Wang G. D. Duan Q. Liu J. (2019). LncRNA MALAT1 facilities high glucose induced endothelial to mesenchymal transition and fibrosis via targeting miR-145/ZEB2 axis. *Eur. Rev. Med. Pharmacol. Sci.* 23 3478–3486. 31081103 10.26355/eurrev_201904_17713

[B22] Liu J. Ji Y. Ai H. Ning B. Zhao J. Zhang Y. (2016). Liver shear-wave velocity and serum fibrosis markers to diagnose hepatic fibrosis in patients with chronic viral hepatitis B. *Korean J. Radiol.* 17 396–404. 10.3348/kjr.2016.17.3.396 27134527 PMC4842858

[B23] Mann J. Mann D. A. (2009). Transcriptional regulation of hepatic stellate cells. *Adv. Drug Delivery Rev.* 61 497–512. 10.1016/j.addr.2009.03.011 19393271

[B24] Monga S. P. (2015). beta-catenin signaling and roles in liver homeostasis, injury, and tumorigenesis. *Gastroenterology* 148 1294–1310. 10.1053/j.gastro.2015.02.056 25747274 PMC4494085

[B25] Popov Y. Schuppan D. (2009). Targeting liver fibrosis: strategies for development and validation of antifibrotic therapies. *Hepatology* 50 1294–1306. 10.1002/hep.23123 19711424

[B26] Pu S. Y. Li Y. P. Liu Q. H. Zhang X. Chen L. Li R. (2021). Inhibition of 5-lipoxygenase in hepatic stellate cells alleviates liver fibrosis. *Front. Pharmacol.* 12:628583. 10.3389/fphar.2021.628583 33679410 PMC7930623

[B27] Puche J. E. Saiman Y. Friedman S. L. (2013). Hepatic stellate cells and liver fibrosis. *Compr. Physiol.* 3 1473–1492. 10.1002/cphy.c120035 24265236

[B28] Rachfal A. W. Brigstock D. R. (2003). Connective tissue growth factor (CTGF/CCN2) in hepatic fibrosis. *Hepatol. Res.* 26 1–9. 10.1016/s1386-6346(03)00115-3 12787797

[B29] Zhang C. H. Wang Y. F. Chen H. Yang G. Wang S. P. Jiang M. N. (2013). Protective effect of the herbal medicine Gan-fu-kang against carbon tetrachloride-induced liver fibrosis in rats. *Mol. Med. Rep.* 8 954–962. 10.3892/mmr.2013.1587 23857550

[B30] Zhang C. Liu X. Q. Sun H. N. Meng X. M. Bao Y. W. Zhang H. P. (2018). Octreotide attenuates hepatic fibrosis and hepatic stellate cells proliferation and activation by inhibiting Wnt/beta-catenin signaling pathway, c-Myc and cyclin D1. *Int. Immunopharmacol.* 63 183–190. 10.1016/j.intimp.2018.08.005 30098497

[B31] Zhang K. Han X. H. Zhang Z. Zheng L. N. Hu Z. M. Yao Q. B. (2017). The liver-enriched lnc-LFAR1 promotes liver fibrosis by activating TGF beta and Notch pathways. *Nat. Commun.* 8:144. 10.1038/s41467-017-00204-4 28747678 PMC5529527

[B32] Zhou X. Yu J. Li Q. Qian W. Xu K.-S. (2008). Effects of transforming growth factor-beta 3 gene transfer on type I collagen synthesis of hepatic stellate cells. *Zhonghua Gan Zang Bing Za Zhi Zhonghua Ganzangbing Zazhi Chinese J. Hepatol.* 16 43–48. 18226343

