# Supplementary material for: Long Noncoding RNA Metastasis-Associated Lung Adenocarcinoma Transcript 1 in Extracellular Vesicles Promotes Hepatic Stellate Cell Activation, Liver Fibrosis and β-Catenin Signaling Pathway
Source: Front Physiol. 2022 Feb 14;13:792182. doi: 10.3389/fphys.2022.792182 (PMC8882958; doi:10.3389/fphys.2022.792182)
Supplement: Supplementary file 1 [file Data_Sheet_1.docx]

**Lnc-MALAT1 in extracellular vesicles promotes hepatic stellate cell activation and liver fibrosis and regulates the β-catenin signaling pathway**

Tianqi Wang, Chong Zhang, Xiaoming Meng, Benshuai Zhu, Siyu Wang, Wenkang Yuan, Sumei Zhang, Jiegou Xu and Chao Zhang

This supplemental material contains 2 supplemental tables and 3 supplemental figures:

Table S1 Primer sequences and siRNA sequences;

Table S2 Clinical characteristic of the patients;

Figure S1. Identification of fibrosis in the liver tissue;

Figure S2. Identification of isolated plasma EVs;

Figure S3. Up-regulated fibrogenesis and β-catenin signaling pathway in LX-2 cells by TGF-β1.

| **Gene** | **Forward sequence** | **Reverse sequence** |
| --- | --- | --- |
| **Collagen I** | 5′-CACCAATCACCTGCGTACAG-3′ | 5′-GCAGTTCTTGGTCTCGTCAC-3′ |
| **α-SMA** | 5′-TCATGGTCGGTATGGGTCAG-3′ | 5′-CCGTGCTCGATAGGGTACTT-3′ |
| **β-catenin** | 5′-TGTTCAGCTTCTGGGTTCAG-3′ | 5′-TATACCACCCACTTGGCAGA-3′ |
| **CyclinD1** | 5′-GCATCTACACCGACAACTCC-3′ | 5′-GATGATCTGTTTGTTCTCCTCC-3′ |
| **c-myc** | 5′-ATCCCTAACTCTACATCAACCC-3′ | 5′-TTCAAATCTCGCTTCCACTT-3′ |
| **GAPDH** | 5′-CTGCCTCGATGGGTGGAGTC-3′ | 5′-AGGCGCCCAATACGACCAAA-3′ |
| **Lnc-MALAT1** | 5′-AGACAGCAGCAGACAGGA-3′ | 5′-AGCTTCCTTCACCAAATCGC-3′ |
| **SiRNA-Lnc-MALAT1** | GCUUAGUUGGUCUACUUUAAA | UAAAGUAGACCAACUAAGCGA |

**Table S1** Primer sequences and siRNA sequences.

**Table S2** Clinical characteristic of the patients.

|  | **Fibrosis**  (n=60) | **High fibrosis**（n=21） | **Low fibrosis**（n=39） | **Control**（n=46） | ***P1* value** | | ***P2* value** |
| --- | --- | --- | --- | --- | --- | --- | --- |
| Gender (M/F) | 36/24 | 12/9 | 24/15 | 19/27 | | 0.0774 | 0.7871 |
| Age | 44.6±9.81 | 50.42±13.76 | 41.46±9.81 | 55.04±13.89 | <0.001 | | 0.0049 |
| BMI (kg/m2) | 22.60±3.62 | 22.95±4.43 | 22.4±3.14 | 22.64±3.08 | 0.9435 | | 0.5747 |
| ALT (U/L) | 64.28±79.08 | 79.8±108.89 | 55.92±57.13 | 25.08±13.23 | <0.001 | | 0.2681 |
| AST (U/L) | 52.98±57.57 | 82.9±84.49 | 36.87±25.04 | 25.54±18.29 | <0.001 | | 0.0024 |
| ALB (g/L) | 41.10±7.48 | 37.33±9.02 | 43.12±5.65 | 43.05±5 | 0.0058 | | 0.0034 |
| TBIL (μmol/L) | 39.42±82.26 | 83.51±128.69 | 15.68±11.69 | 15.62±15.9 | <0.001 | | 0.0017 |
| HBeAg (+/-) | 17/43 | 6/15 | 11/28 | 8/38 | 0.2497 | | >0.9999 |

P1 values, fibrosis group vs control group.

P2 values, high fibrosis group vs low fibrosis group.


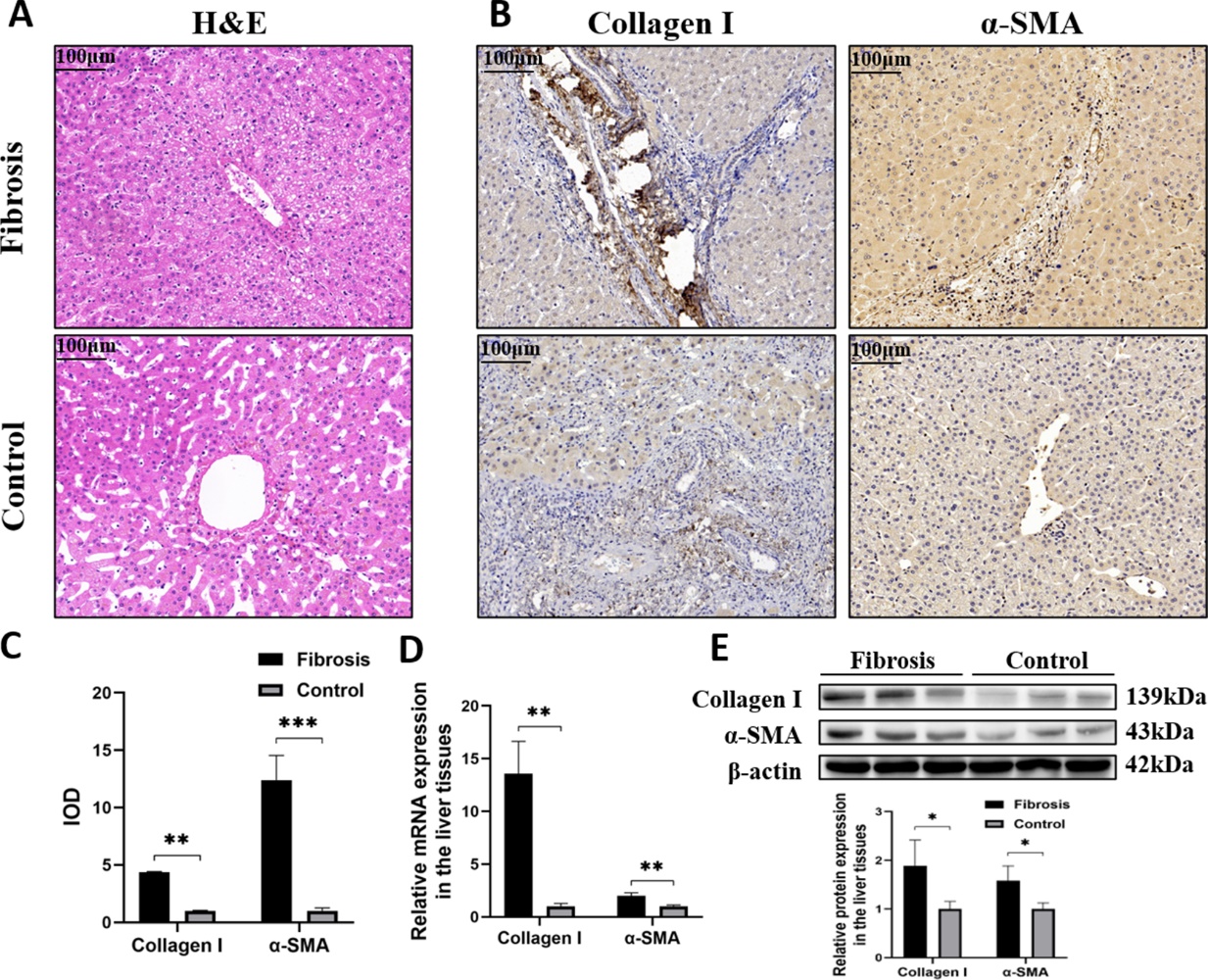


**Figure S1.** Identification of fibrosis in the liver tissue.

Dissected liver tissues from liver fibrosis patients and the control patients were used for pathological examination, qRT-PCR and western blot analyses. (A) representative images of HE staining. (B) representative images of IHC for collagen Ⅰ and a-SMA. (C) IOD values of collagen I and α-SMA measured by Image-pro plus (n=6). (D) relative mRNA expression of α-SMA and Collagen I detected by qRT-PCR (n=6). (E) The protein expression of α-SMA and collagen I detected by western blots (n=6). *, ** and *** represent *p* values less than 0.05, 0.01 and 0.001, respectively.


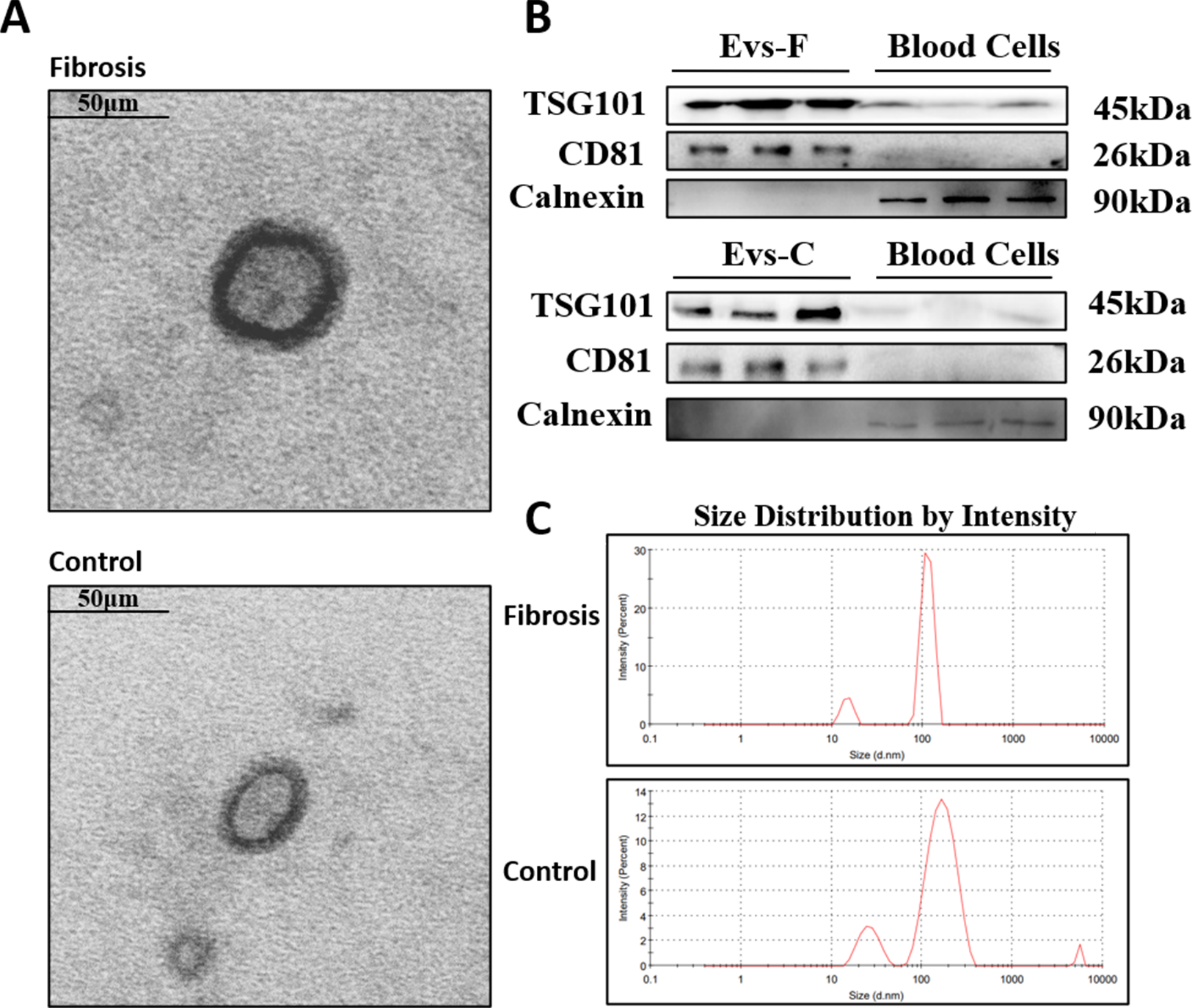


**Figure S2.** Identification of isolated plasma EVs.

EVs were isolated from the plasma of liver fibrosis patients and identified for their structure, components and size. (A) TEM observation of the isolated EVs. The fibrosis patient group is at the top, and control group is at the bottom. (B) EVs (n=6) western blot analysis of TSG101, CD81 and Calnexin, with blood cell lysate as a negative control (n=6). (C) Nano Particle Tracking analysis of the size distribution of the EVs.


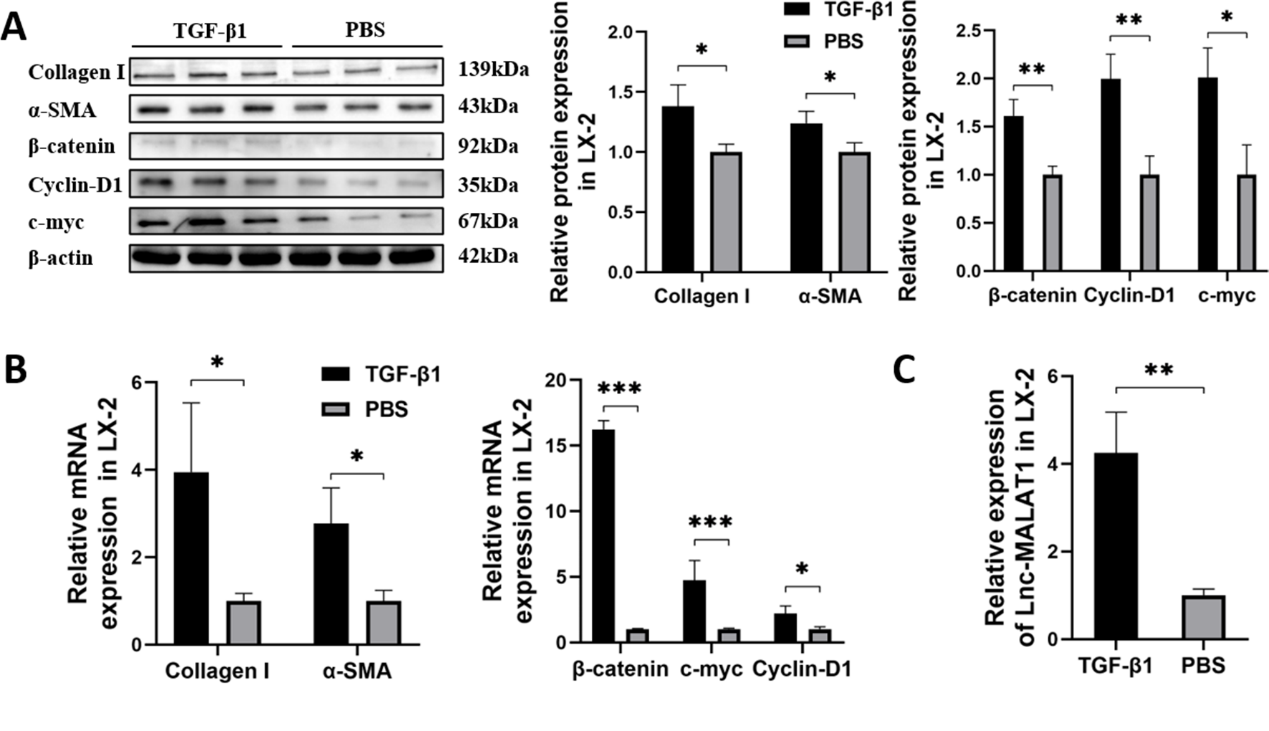


**Figure S3.** Up-regulated fibrogenesis and β-catenin signaling pathway in LX-2 cells by TGF-β1.

1×10^6^ LX-2 cells in a 6-well plate were treated with 10ng/ml TGF-β1 or equivalent volume of PBS for 48 hours. The cells were harvested for western blot and qRT-PCR analyses. (A) the protein level of α-SMA, collagen I, β-catenin, cyclin-D1 and c-myc was detected by western blot and quantified by ImageJ (n=6). (B) the relative mRNA expression of α-SMA, collagen I, β-Catenin, cyclin-D1 and c-myc was detected by qRT-PCR (n=6). (C) the relative expression of Lnc-MALAT1 was examined (n=6). *, ** and *** represent *p* values less than 0.05, 0.01 and 0.001, respectively.
